# Expression of podoplanin in salivary gland adenoid cystic carcinoma and its association with distant metastasis and clinical outcomes

**DOI:** 10.3892/mmr.2012.911

**Published:** 2012-05-09

**Authors:** HE-MING WU, GUO-XIN REN, LI-ZHEN WANG, CHUN-YE ZHANG, WAN-TAO CHEN, WEI GUO

**Affiliations:** 1 Department of Oral and Maxillofacial - Head and Neck Oncology, Ninth People’s Hospital, School of Medicine, Shanghai Jiao Tong University; 2 Shanghai Key Laboratory of Stomatology, Department of Oral and Maxillofacial - Head and Neck Oncology, Ninth People’s Hospital, School of Medicine, Shanghai Jiao Tong University; 3 Department of Oral Pathology, Ninth People’s Hospital, Shanghai Jiao Tong University School of Medicine, Shanghai 200011, P.R. China

**Keywords:** podoplanin, D2-40 antibody, salivary gland adenoid cystic carcinoma, prognostic marker, distant metastasis

## Abstract

Distant metastasis is a common cause of mortality in patients with salivary gland adenoid cystic carcinoma (SACC). However, presently, the development of distant metastasis is unable to be predicted in clinical practice. Recent studies have shown that overexpression of podoplanin is associated with metastasis and survival in patients with several cancer types. The purpose of the present study was to determine whether podoplanin is overexpressed in SACC and whether such overexpression is associated with distant metastasis and survival. Podoplanin expression was determined using immunohistochemistry (IHC) in tumors from 40 SACC patients. The expression status was analyzed in regards to patient clinicopathological parameters and survival rates. Overexpression of podoplanin was detected in 13 (32.5%) of the 40 tumors. Overexpression was significantly associated with disease-free survival (P=0.025) and distant metastasis (P=0.015), although it was not associated with recurrence and overall survival. In conclusion, podoplanin is overexpressed in a subset of SACCs and may be a biomarker predicting distant metastasis in patients with SACC.

## Introduction

Adenoid cystic carcinoma (ACC), one of the most common malignant tumors of the major and minor salivary glands, displays certain unique characteristics, such as slow but aggressive growth, strong invasion to peripheral nerves or blood vessels at the early phase, and a high incidence of recurrence and distant metastasis (1,2). The incidence of distant metastasis (mostly in lungs) for patients with salivary gland adenoid cystic carcinoma (SACC) ranges from 35 to 50%, whereas lymph node metastases are rare. Although the 5-year survival rate is approximately 70% for patients with SACC, the survival rates decrease to 40% at 10 years and 25% at 15 years due to frequent local recurrence and distant metastasis (3–8). The current strategy to reduce local recurrence is postoperative radiotherapy. It has been suggested that neoadjuvant or adjuvant chemotherapy may reduce the incidence of distant metastasis and improve the disease-free survival of patients (2,3). Clinicopathological characteristics are the major factors used to evaluate the risk of distant metastasis of SACC. For example, a significantly higher incidence of distant metastasis occurs in patients with solid histology subtype (6,7). The size of primary tumors has also been associated with the incidence of distant metastasis (7). However, controversial reports have been published in the literature (3–7). The molecular mechanism involved in the development and progression of SACC remains uncertain, yet a strong prognostic indicator is demanded for the appropriate choice of therapy for SACC patients.

Podoplanin (also termed as T1a-2, aggrus or gp36) is a small mucin-like protein and its physiological function is related to tissue development and repair (9,10). It is highly and specifically expressed in lymphatic endothelial cells while not found in blood endothelium (9). Therefore, podoplanin is widely used as a specific marker for lymphatic endothelial cells and lymphangiogenesis in many species. Recent studies, however, have shown that podoplanin is also expressed in certain tumor cells, including many central nervous system, cervix, germinal and mesothelioma tumors, as well as oral squamous cell carcinoma (11–15). For cervix and oral squamous cell carcinoma, podoplanin is associated with migration/invasion, making it a novel prognostic marker for patients with these tumors (11,12).

In this study, our team used monoclonal antibody D2-40, which specifically recognizes podoplanin, to determine the expression patterns of this protein in patients with primary SACC. Moreover, we analyzed the associations between the expression patterns and clinicopathological characteristics, as well as the clinical outcomes.

## Materials and methods

### Patient population

All 40 patients analyzed in this study were diagnosed with SACC at the Department of Oral and Maxillofacial Surgery, Shanghai Ninth People’s Hospital, between January 1999 and December 2003, and underwent complete surgical resection with curative intent. Of the 40 patients, 22 were females and 18 males, and the median age was 51.9 years (range, 28–76 years). Twenty tumors (50%) originated from the major salivary glands, and 20 tumors (50%) from the minor salivary glands. The tumor classification were based on the histological grading system recommended by Dardick (16): grade I, tumors with cribriform and/or tubular structures without a solid component; grade II, tumors composed of cribriform and/or tubular structures with <30% of solid areas; and grade III, tumors with >30% of solid areas. The clinical staging of the patients was determined according to criteria set in the Tumor, Node, Metastasis (TNM) system of the International Union Against Cancer, 2000 (17). Tumor recurrence and metastasis were confirmed by radiographical and pathological diagnosis. The follow-up period was 6–101 months (mean, 64.95 months). Of the 40 patients, 3 had enlarged cervical lymph nodes, 11 had tumor recurrence and 16 had distant metastasis. Eight (20%) of the 40 patients died of recurrence or metastasis. All patients signed an informed consent form prior to enrollment in the study, and permission was obtained from the ethical committee of the Shanghai Ninth People’s Hospital.

### Tumor tissues and immunohistochemistry (IHC)

Formalin-fixed, paraffin-embedded tissues were cut into 4-μm sections, mounted on glass slides, and then deparaffinized in graded alcohol. Endogenous peroxidase activity was blocked with 3% H_2_O_2_ for 20 min. The slides were incubated overnight at 4°C with the primary antibody D2-40 (mouse monoclonal, 1:20 dilution; AbD Serotec, MorphoSys UK Ltd., Oxford, UK). The primary antibody binding was detected using peroxidase labelled secondary antibody and chromogen, diaminobenzidine (DAB) Dako EnVision™ Detection Systems (Dako, Denmark) according to the manufacturer’s recommendations. Tissue sections were counterstained with hematoxylin. Negative controls were treated using the same procedures but omitting the use of the primary antibody. The staining of adjacent lymphatic endothelial cells within the same sections was used as the positive internal control. Negative controls were prepared by replacing the primary antibodies with non-immune mouse serum, and no reactive products were detected.

Cytoplasmic and/or membrane immunoreactivity were considered to indicate podoplanin expression positivity. All the slides were reviewed independently by 2 pathologists (L.Z.W. and C.Y.Z.) without the knowledge of the clinical information of the patients. The slides were evaluated using a microscope with a square grid (10×10 mm) in the ocular lens. Under magnification of ×40 to delineate the area, each case was evaluated in terms of staining intensity, and the number of positive and negative tumor cells in at least 4 fields was quantified. Scores were based on staining extention: no positive tumor cells, 0; <10% positive tumor cells, 1; 10–30% positive tumors cells, 2; >30% positive tumors cells, 3. A score of 0 and 1 was considered low reactivity, while >1 was considered high.

### Statistical analysis

Statistical tests were performed using SPSS software (version 11.0, SPSS Inc, Chicago, USA). The relationship between podoplanin expression and clinicopathological parameters was determined using Wilcoxon test amd signed rank sum tests. Fisher’s exact probability test was used to analyze the statistical significance. Overall survival time was calculated from the date of initial diagnosis to death or the last day of follow-up evaluation. The period from the date of therapy to the date of recurrence or metastasis was considered as disease-free survival. Survival analysis was computed by means of the Kaplan-Meier method, and levels of significance were assessed by means of the log-rank test. P-value <0.05 was considered to indicate a statistically significant difference.

## Results

### Podoplanin expression and clinicopathological features

**Figure 1:**
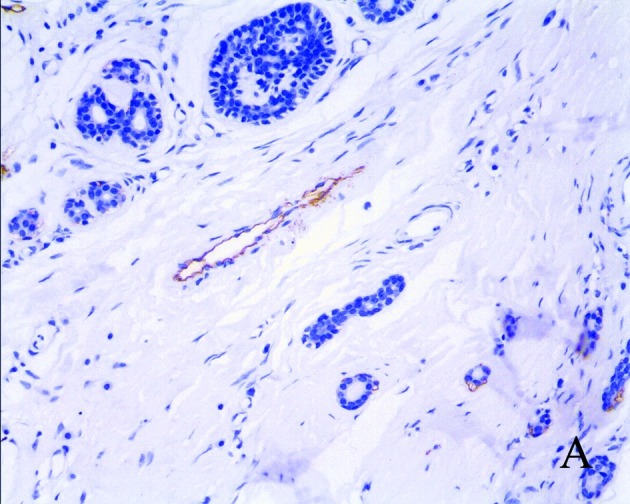

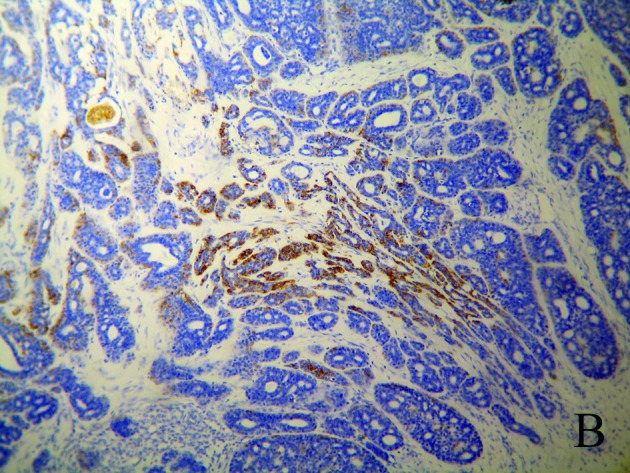
(A) Podoplanin expressed in the lymphatic endothelial cell (magnification, ×200). (B) Podoplanin expressed in salivary gland adenoid cystic carcinoma (SACC) (magnification, ×40).

As expected, podoplanin was highly expressed in the endothelial cells of lymphatic vessels (Fig. 1A). Normal salivary glands around the tumor revealed a positive cytoplasmic and/or membrane reaction with the podoplanin antibody; the positive cells were mainly located in myoepithelial cells and various zones of the secretory duct system.

Among the 40 tumors analyzed, 9 (22.5%) did not demonstrate podoplanin expression and 18 (45%) had <10% positive tumor cells, which were considered to have low expression. The other 13 samples (32.5%) were considered to have high expression, of which 8 had 10–30% positive tumor cells, while 5 had >30% positive tumor cells (Fig. 1B). The relationship between podoplanin expression and clinicopathological features is summarized in Table I. Of the 13 specimens with high positive podoplanin staining, 9 patients developed distant metastasis. Of the remaining 27 patients with low expression, 7 had distant metastasis. Statistical analysis indicates that positive podoplanin staining is significantly correlated with distant metastasis (P=0.015, n=40). There was no significant difference between the high and low podoplanin expression group with respect to age at diagnosis, gender, T-classification tumor histological grading, regional metastasis and recurrence. However, podoplanin expression was higher in tumors located in the sublingual gland than that in tumors located in other glands.

### Podoplanin expression and survival

**Figure 2:**
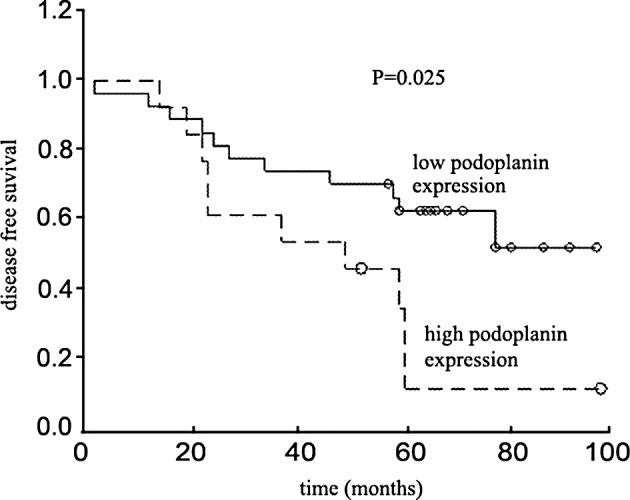
Disease-free survival rate of patients with salivary gland adenoid cystic carcinoma (SACC), (n=40).

Although patients whose tumors expressed high levels of podoplanin had a shorter overall survival compared to those whose tumors expressed low levels of podoplanin, the difference was not statistically significant (P=0.789, log-rank test). However, the difference in disease-free survival was statistically significant (P=0.025, log-rank test) (Fig. 2). Fig. 2 depicts the Kaplan-Meier plot of the survival curves.

## Discussion

The monoclonal antibody D2-40 was initially developed to recognize the M2A antigen, which is an oncofetal glycoprotein expressed by testicular germ cell neoplasms (11). According to a recent study carried out using multiple approaches, the D2-40 antibody specifically recognizes human podoplanin, which has biochemical characteristics similar to the M2A antigen (11). In normal human tissue, podoplanin is expressed in kidney podocytes, skeletal muscle, placenta, lung and heart, myoepithelial cell of the breast and salivary glands, osteoblasts and mesothelial cells (10). Although the biological functions of podoplanin are not fully understood, it may promote the formation of elongated cell extensions and increase adhesion, migration, and tube formation of vascular endothelial cells. ERM-protein phosphorylation may link podoplanin expression to the observed rearrangement of the actin cytoskeleton (15).

In the mouse salivary gland, podoplanin was found to be a salivary gland myoepithelial cell antigen, and the detection level directly reflects myoepithelial cell distribution (18). Strong expression of podoplanin is also found at the basal portion of the intercalated, striated and interlobular ducts (19). Recently, Fujita *et al* found that various SACC tumor cells or ‘neoplastic myoepithelial cells’ are podoplanin-positive (20). In the present research, the overall expression level of podoplanin in SACC tumor cells was low (only 13 samples had more than 10% expression). However, approximately 70% (9 of 13) of patients with podoplanin expression developed distant metastasis, significantly higher than the occurrence in the patients with weak or no podoplanin expression. Importantly, the disease-free survival rate was significantly lower. These data suggest the potential use of podoplanin as a molecular maker for distant metastasis in SACC. In mouse salivary gland, podoplanin expression was rarely found in acini of the parotid glands, while it was clearly found in the basal portion surrounding acinar cells of the submandibular and particularly the sublingual glands (19). Regarding the high expression in sublingual glands, our results suggest that podoplanin overexpression may be dependent on the gland involved. Nevertheless, further studies with larger sample sizes are needed to validate our findings. Furthermore, the detailed mechanisms underlying the relationship between high podoplanin expression and distant metastasis in SACC warrant further investigation.

Nevertheless, of the 16 patients that developed distant metastasis, 7 did not exhibit high expression of podoplanin, indicating the limitation of using podoplanin as a marker of distant metastasis. Additional molecular markers are desirable to compensate for the limitation of podoplanin.

In summary, our results revealed the differential expression of podoplanin among SACC cases. There was a close correlation between podoplanin expression and distant metastasis and disease-free survival. Further study is needed to elucidate the main role of podoplanin in tumorigenesis and the biological behaviors of SACC.

## Figures and Tables

**Table I:** Correlation between podoplanin expression and patient characteristics.

	Low podoplanin expression (n=27)	High podoplanin expression (n=13)	P-value
Age at diagnosis			
<60 years	18	10	0.716
≥60 years	9	3	
Gender			
Female	17	5	0.314
Male	10	8	
T classification			
I or II	15	5	0.501
III or IV	12	8	
Regional metastasis			
With	3	0	0.538
Without	24	13	
Distant metastasis			
With	7	9	0.015
Without	20	4	
Recurrence			
With	7	4	1.000
Without	20	9	
Histological grading			
I	11	5	0.644
II	13	5	
III	3	3	
SACC tumor location (gland)			
Parotid	4	0	0.199
Submaxillary	4	1	
Sublingual	5	6	
Small	14	6	

SACC, salivary gland adenoid cystic carcinoma.
